# Targeting brain estrogen receptor for binge eating

**DOI:** 10.18632/oncotarget.5239

**Published:** 2015-08-22

**Authors:** Pingwen Xu, Xuehong Cao, Yong Xu

**Affiliations:** Children’s Nutrition Research Center, Department of Pediatrics and Department of Molecular and Cellular Biology, Baylor College of Medicine, Houston, TX, USA

**Keywords:** BED, estrogen, serotonin

Binge eating disorder (BED) is characterized by the ingestion of a large amount of food in a short period of time and the preferential choice of high palatable food. BED is the most common eating disorder, which is associated with other medical and psychiatric complications, most notably obesity and depression [1]. The effective treatment for BED is urgently needed.

We adapted a recently developed training paradigm that effectively induces a sustained binge-like eating behavior in mice, which mimics many characteristics of binge eating in BED patients [2]. We first showed that estrogen replacement substantially suppresses binge-like eating behavior in ovariectomized female mice [3], which is consistent with the previous reports that binge eating in women is negatively associated with circulating estrogen levels [4]. Our results prove the principle that estrogen replacement therapy could be used to ameliorate binge symptoms in women. However, the current estrogen replacement therapy is often associated with detrimental side effects, e.g. breast cancer. One solution for this dilemma is to understand where and how the estrogen acts to inhibit binge-like eating behavior, which may facilitate the development of more specific anti-binge estrogen-based therapy that could avoid the side effects.

Selective serotonin reuptake inhibitors, which enhance brain 5-hydroxytryptamine (5-HT) signals, showed some anti-binge efficacy in clinical trials [5]. Interestingly, estrogen stimulates central 5-HT neuron activities [6]. These findings led to a possibility that estrogen acts upon brain 5-HT neurons to inhibit binge eating. Current development of transgenic mouse lines makes it possible for us to definitively test physiological roles of complex neural networks in binge behavior. We showed that the estrogen-induced inhibition on binge-like eating was blocked in female mutant mice lacking estrogen receptor-α (ERα) only in 5-HT neurons in dorsal raphe nuclei (DRN), indicating that estrogen acts through ERα expressed by DRN 5-HT neurons to inhibit binge-like eating behavior. Through electrophysiological recordings in identified 5-HT neurons, we confirmed that propylpyrazole triol (PPT, an ERα agonist) depolarizes DRN 5-HT neurons and stimulates their firing activities. We further demonstrated that a small conductance Ca2+-activated K+ (SK) current is required for the stimulatory effects of PPT on DRN 5-HT neurons. More importantly, local inhibition of the SK current in the DRN remarkably suppressed binge-like eating in female mice. Collectively, our results support a model that estrogen acts upon ERα to inhibit the SK current in DRN 5-HT neurons thereby activating these neurons to suppress binge-like eating behavior. These findings identify ERα and/or SK current in DRN 5-HT neurons as potential targets for anti-binge therapies.

Given the fact that estrogen replacement therapy is associated with increased risk of breast cancer, our collaborator, Dr. DiMarchi from Indiana University, developed an estrogen-based conjugate named glucagon-like peptide-1-estrogen (GLP-1-estrogen). This conjugate uses GLP-1 as a “carrier” to deliver estrogens preferentially to GLP-1 receptor-enriched regions, which can produce profound body weight lowering effects but with minimal uptake by the breast tissue [7]. We found that this conjugate effectively delivers bioactive estrogen to the DRN and substantially suppressed binge-like eating in ovariectomized female mice. Together, our studies not only demonstrated a mechanism by which estrogen may regulate binge eating, but may also provide a strong case for a new compound that targets ERα in the DRN 5-HT neurons to treat binge eating.

Future directions include delineating the intracellular mechanisms by which estrogen regulates 5-HT neurons, and identifying the neural circuits downstream of 5-HT neurons that are important for the regulation of binge-like eating behavior. We hope these efforts will identify more rational targets for the development of novel therapies that can treat BED and other related eating disorders.
